# A Dual-Weighting and Adaptive Regularization Framework for Takagi–Sugeno Identification of Nonlinear Dynamic Systems from Closed-Loop Sensor Data

**DOI:** 10.3390/s26206646

**Published:** 2026-10-21

**Authors:** Gabriel Moreano, Julio Tafur, Valeria Andino, Franklin Sánchez

**Affiliations:** 1 Department of Electrical, Electronics and Telecommunications, Universidad de las Fuerzas Armadas ESPE, Av. General Rumiñahui S/N, Sangolquí 171103, Ecuador; fasanchez@espe.edu.ec; 2 Department of Engineering, Pontificia Universidad Católica del Perú (PUCP), Lima 15088, Peru; jtafur@pucp.edu.pe; 3 Faculty of Business Administration, Escuela Superior Politécnica de Chimborazo (ESPOCH), Riobamba 060155, Ecuador; valeria.andino@espoch.edu.ec

**Keywords:** Takagi–Sugeno models, closed-loop identification, fuzzy systems, weighted least squares, adaptive regularization, sensor noise

## Abstract

In sensor-based monitoring and control systems, dynamic models are often identified from data collected in closed loop (where the noisy sensor measurements that drive the controller are also regressors). Feedback links regressors to disturbances, which violates one of the basic assumptions of least squares and leads to biased—and sometimes highly unstable—estimates. This difficulty is worsened in Takagi–Sugeno (TS) models, as they involve global coupling: poorly excited regions can dominate the model. In this work, we address this by proposing Dual-Weighted Takagi–Sugeno Identification (DW-TSI), a framework that combines dual weighting with adaptive regularization. We achieve this through a rule-wise weighted least-squares formulation that decomposes the global problem into local subproblems, weighting each sample by its membership activation and preserving the interpretability of the local submodels in state space; an adaptive ridge term then scales the penalty of each rule inversely with its cumulative activation, ensuring that conditioning is reinforced exactly where the sensor data are weakest. The framework is extensively validated on an open-loop unstable physical aero-pendulum, identified using only closed-loop angular measurements. Unlike classical TS models, DW-TSI maintains boundedness in a multi-step autonomous simulation, and it approximates subspace identification (N4SID) accurately. A sensor noise injection study further shows that the proposed estimator degrades gracefully in the presence of measurement noise, whereas unregularized TS estimation collapses. These models can be deployed for sensor-based feedback control without any retuning.

## 1. Introduction

In many of today’s control and sensor-driven applications, from robot dynamics [1,2,3] to biochemical system behavior [4,5,6], such modeling of nonlinear dynamic systems is central since local linear approximations do not suffice while behavior is strongly state-dependent. With the proliferation of sensing and embedded computing, these systems are more frequently monitored and controlled using streams of noisy sensor data, leading to an ever-increasing need for accurate empirical models based on data. Of all the representations, one of particular importance is Takagi–Sugeno (TS) fuzzy models; TS fuzzy models represent nonlinear dynamics as a smooth interpolation among linear submodels that span an operating region [7,8,9,10].

A TS structure manifests a nonlinear system in terms of a fuzzy rule base, which associates a subregion of the state space with its linear model. This results in a concise yet informative form whereby the global nonlinearity is represented at run time through membership functions that modulate the contribution from each submodel on the fly [11,12]. This is how TS models leverage the interpretive power of rule-based systems and the analytical tractability of linear systems.

A key benefit of this representation is that it provides a formal mechanism to extend linear analysis and control-design tools—such as state feedback or parallel distributed compensation (PDC)—to nonlinear systems, while keeping stability and performance properties verifiable [13,14,15]. Consequently, the flexibility and performance of TS models have made them a de facto standard for the control of nonlinear systems.

On the other hand, the quality of a TS model depends on how the identification process is used to derive it from data. Well-defined membership functions, along with consistent physically interpretable local submodels, will maintain structural fidelity. Nevertheless, notwithstanding the representational strengths of the TS framework, it is still not straightforward to obtain such models from experimental data under realistic operating conditions [16,17,18,19].

It is in fact an identification problem, typically estimating a set of parameters from a structural description relating the variables being measured [20,21,22]. Under some formulations, it is possible to cast this relationship as a linear regression in the unknown parameters and apply least-squares methods directly to obtain optimal estimates with respect to mean squared error.

Under the best circumstances—for a persistent excitation and statistical independence between the regressors and disturbances—the least-squares estimates are consistent and efficient [23,24]. However, these assumptions are rarely satisfied in practice, particularly when data are collected under feedback [25,26]. To the point that if they fail, it undermines both the foundations of the method and the reliability of its estimates.

Statistical independence between the regressors and the system disturbances is a minimal requirement for the validity of least-squares estimators [27,28]. The exogeneity condition: the prediction error is to be uncorrelated with the information used to estimate the parameters, which is necessary for consistency.

In practice, the availability of open-loop data is limited [29], and the process data acquired online are not independent of an active controller [30,31,32]. The input here is not externally driven nor entirely independent, it arises from feedback defined by the sensor observations provided by the system itself. Since these measurements are always contaminated by sensor noise and disturbances, an indirect dependence then exists between the input applied and the disturbances acting on the system in a closed-loop sense.

This correlation leads to bias or systematic error in the parameter estimates and makes it impossible for ordinary regression methods (even with a lot of data) to discover the dynamical system accurately.

The issue is more prominent in nonlinear systems, where the variable interactions are complex and can inadvertently generate high correlation through themselves [32,33,34,35]. This bias is especially detrimental for identification schemes which need to provide invariance across multiple local operating regimes, which is the situation we face with TS models.

Many approaches have been proposed to tackle closed-loop identification in the linear setting by reformulating the problem with various methods to establish consistency, such as instrumental variables [36,37,38,39,40], error models [41,42,43,44], or through subspace state-space identification [45,46,47,48]. In contrast, for nonlinear dynamical systems, these time-domain formulations are rarely possible and typically rely on model specific assumptions and expansions (e.g., Gaussian) rather than common fixed basis functions. However, these methods produce internal representations that are somewhat abstract without recognizable physical meaning [49,50].

While this is sufficient for many prediction or control tasks [51,52,53], such representations are ill-suited to building structured models based on physically interpretable states.

The TS formulation has a limit of partitioning that reflects the physical variables that influence the state system dynamics. In cases where the states employed share no physical meaning, the definition of membership functions and interpretation of fuzzy rules become meaningless and undermine model structural validity.

Despite substantial progress in both system identification and Takagi–Sugeno modeling, no existing framework builds TS models from closed-loop data while respecting the local nature of the submodels and the physical meaning of their states.

These strategies do not either ensure the compatibility of local submodels in feedback or framework how the induced correlation is affected by the estimates. This points towards the fact that we could still use a framework to counteract closed-loop bias, while maintaining this important local structure of TS models.

In this work, we propose Dual-Weighted Takagi–Sugeno Identification (DW-TSI), a framework for the identification of Takagi–Sugeno models based on closed-loop sensor data. This name comes from the two complementary weightings, both defined by the rule activations: the first is for the residuals of the local cost function and another for the regularization penalty of each rule. We summarize the contributions as follows:

1. Weighted least-squares-based estimator, where we extend our formulation to a rule-wise weighted least-squares estimator that directly incorporates the fuzzy membership structure into the estimation process, which allows decoupling a large-scale global problem into local subproblems while each state-space submodel preserves its physical interpretability.
2. We propose an adaptive regularization strategy that increases the penalty inversely as a function of the cumulative activation, which assists with numerical conditioning and estimation in weakly excited operating regions.
3. In this work, we analyze a theoretical grounding showing how our formulation decouples the rule-wise subproblems and converges to global ridge regression in the uniform activation regime, as well as strictly improving the conditioning of each local information matrix, by providing a structural mechanism restricting feedback-based correlation to the subregion where each rule is active.
4. We demonstrate the applicability of our framework on a physical aero-pendulum identified only from closed-loop data and test the robustness of this system with a sensor noise injection study, whose results show that the proposed estimator degrades gracefully while unregularized TS estimation collapses.

The rest of the article is structured as follows: Section 2 reviews the foundations of Takagi–Sugeno modeling and its interpretation as a linear regression problem, highlights limitations of global least squares in TS models and lays out the closed-loop identification problem (Section 2.4), including ensuing violations of some least-squares assumptions leading to bias. In Section 3, we propose our framework with the rule-wise weighted estimator and adaptive regularization strategy, together with all theoretical analysis. The experimental aero-pendulum platform and fuzzy partition are described in Section 4, as well as the performance of the proposed approach compared to classical TS and N4SID varying from one-step-ahead prediction, multi-step autonomous simulation and closed-loop applicability tasks, downstream on a sensor noise injection test. The findings and limitations are presented in Section 5, followed by the paper conclusion in Section 6.

## 2. Background

### 2.1. TS Model Representation

A discrete-time Takagi–Sugeno (TS) model can be represented as a set of *r* fuzzy rules of the following form:

(1) $$R_{i}:\;\mathrm{If}\;x_{k}\in X_{i},\;\mathrm{then}\;x_{k+1}=A_{i}x_{k}+B_{i}u_{k},$$

for i=1,…,r, where $x_{k}\in R^{n}$ denotes the system state, $u_{k}\in R^{m}$ is the input, and $A_{i}$ and $B_{i}$ define the parameters of the local submodel associated with the *i*-th fuzzy rule.

Membership functions in the state space determine if each rule is activated. Let $\mu_{i}\left(x_{k}\right)$ be the degree of activation of rule *i*, then the normalized weight can be expressed as

(2) $$w_{i}\left(x_{k}\right)=\frac{\mu_{i}\left(x_{k}\right)}{\sum_{j=1}^{r}\mu_{j}\left(x_{k}\right)}$$

where $w_{i}\left(x_{k}\right)\in \left[0,1\right]$ and $\sum_{i=1}^{r}w_{i}\left(x_{k}\right)=1$, only when there is a partition of unity among the sets.

The overall system dynamics are derived through a convex combination of the local submodels:

(3) $$x_{k+1}=\sum_{i=1}^{r}w_{i}\left(x_{k}\right)\left(A_{i}x_{k}+B_{i}u_{k}\right)$$

### 2.2. TS Identification as Linear Regression

The Takagi–Sugeno model can be written in an organized manner to reformulate the parameter identification problem as linear regression. This property follows from the system dynamics being linear in the submodel parameters given states and inputs.

Based on the global representation (Equation (3)), where $x_{k}\in R^{n}$, $u_{k}\in R^{m}$, and $w_{i}\left(x_{k}\right)$ are the normalized weights, the extended variable vector is introduced as

(4) $$\xi_{k}=\left[\begin{array}{c} x_{k}\\ u_{k} \end{array}\right]\in R^{n+m}$$

Under

this formulation, each local submodel can be represented as a linear transformation:

(5) $$\Theta_{i}=\left[\begin{array}{cc} A_{i} & B_{i} \end{array}\right]\in R^{n\times (n+m)}$$

thereby enabling the system dynamics to be reformulated as

(6) $$x_{k+1}=\sum_{i=1}^{r}w_{i}\left(x_{k}\right)\;\Theta_{i}\;\xi_{k}$$

To rewrite this expression in a global regression form, the rule-weighted regressor vector is introduced as

(7) $$\phi_{i,k}=w_{i}\left(x_{k}\right)\;\xi_{k}$$

and the complete regressor vector is then constructed as

(8) $$\phi_{k}=\left[\begin{array}{c} \phi_{1,k}\\ \phi_{2,k}\\ \vdots \\ \phi_{r,k} \end{array}\right]\in R^{r(n+m)}$$

Under these definitions, the system dynamics can be written in the following compact linear regression form:

(9) $$x_{k+1}=\Theta \phi_{k}$$

From a set of *N* experimental samples, the data matrices are constructed as

(10a) $$X^{+}=\left[\begin{array}{cccc} x_{2} & x_{3} & \cdots & x_{N} \end{array}\right]\in R^{n\times (N-1)}$$

(10b) $$\Phi =\left[\begin{array}{cccc} \phi_{1} & \phi_{2} & \cdots & \phi_{N-1} \end{array}\right]\in R^{r(n+m)\times (N-1)}$$

which allows the identification problem to be formulated as

(11) $$X^{+}=\Theta \Phi$$

The estimation of the parameter matrix Θ is obtained by minimizing the squared prediction error measured in the Frobenius norm:

(12) $$J\left(\Theta \right)={∥X^{+}-\Theta \Phi ∥}_{F}^{2}$$

By taking the derivative of this criterion with respect to Θ and setting the gradient to zero, the normal equation is obtained:

(13) $$\Theta \Phi \Phi^{T}=X^{+}\Phi^{T}$$

Provided that the information matrix $\Phi \Phi^{T}$ is nonsingular, the closed-form solution to the least-squares problem is given by

(14) $$\hat{\Theta }=X^{+}\Phi^{T}{\left(\Phi \Phi^{T}\right)}^{-1}$$

This formulation underlies classical TS identification methods where all parameters are estimated jointly from the data.

Many approaches were proposed to solve the problems related to singularity or poor conditioning of the information matrix used in estimators. Perhaps the most commonly used method is matrix regularization (applied via a ridge-like penalty) to keep the matrix invertible [54,55,56]. Moreover, spectral truncation or singular value decomposition (SVD) is also an effective method to remove poorly conditioned regions in the parameter space [6,57,58]. In addition, there are well-studied formulations that use generalized pseudoinverses that can be safely used in the presence of singular matrices.

### 2.3. Limitations of Global LS in TS Models

The identification representation outlined in Section 2 gives rise to a global least-squares estimation of the parameters involved within a Takagi–Sugeno model. The above method has its own flaws when broadly applied to structured models, which contain many local submodels.

The first one would be that components of the global regressor have a high collinearity while constructing. The global regressor has identical blocks of an extended vector in every block (which can result in high column correlation between the columns) scaled to a different activation weight. It also leads to linear dependence in regions where multiple rules ignite similarly. This introduces a little more structural redundancy, which reduces the rank and thus might lead to numerical instabilities of the estimation process.

Second, local submodels are excited in an asymmetric fashion. However, as the data points are not always evenly spread throughout space, some rules end up with too little information. Hence, the parameters associated with those poorly excited regions are inferred with high uncertainty, which results in ill-conditioned directions in the parameter space.

Additionally, global estimation also causes an implicit coupling between the submodels. In this case, all parameters are captured from a unified optimization and thus data across operating regions are blended when estimating. This coupling makes each submodel identifiable individually but the parameters that we identify are less representative of local system dynamics.

These restrictions are characteristic of the standard least-squares formulation when applied to TS models. This renders the numerical conditioning of the problem ill-posed and endangers the local coherence of the model we identified.

Hence, identification methods utilizing the structure of the Takagi–Sugeno model must be developed that decouple the estimation of its submodels and allow for distributions which adapt with respect to rule activations.

### 2.4. Closed-Loop Identification Problem

Consider a dynamical system described by

(15) $$x_{k+1}=f\left(x_{k},u_{k}\right)+\eta_{k}$$

where $x_{k}\in R^{n}$ defines the system state, $u_{k}\in R^{m}$ is the control input, and $\eta_{k}$ is modeled as a disturbance or process-noise term.

Now, note that in a closed-loop identification scheme, $u_{k}$ is not an independent external input, but an internal control law which acts on the system variables. In particular, a linear feedback of the following form is studied:

(16) $$u_{k}=Kx_{k}+r_{k}$$

where $K\in R^{m\times n}$ represents the feedback gain matrix, and $r_{k}$ denotes a reference signal or external excitation.

Substituting the control law into the system dynamics yields

(17) $$x_{k+1}=f\left(x_{k},Kx_{k}+r_{k}\right)+\eta_{k}$$

This demonstrates that the system’s evolution depends on an input directly correlated with its state, meaning that disturbances affect the measurements.

This dependency implies that the variables used for identification $x_{k}$ and $u_{k}$ are not statistically independent of the noise terms. This means that closed-loop data regression creates dependencies that compromise the process.

### 2.5. Violation of LS Assumptions

The validity of the least-squares estimator in Equation (14) relies on the orthogonality between the regressor and the prediction error, namely

(18) $$E[\phi_{k}e_{k}]=0,$$

where E[·] denotes the expectation operator, $\phi_{k}$ is the regressor vector built from the measured system variables, and $e_{k}$ is the prediction error given by

(19) $$e_{k}=x_{k+1}-\Theta \phi_{k}.$$

This condition, however, is not satisfied under closed-loop identification. Using the true system dynamics (Equation (15)) together with the model approximation (Equation (9)), the prediction error becomes

(20) $$e_{k}=f\left(x_{k},u_{k}\right)-\Theta \phi_{k}+\eta_{k}.$$

The regressor $\phi_{k}$ depends on both $x_{k}$ and $u_{k}$. Under the closed-loop input law (Equation (16)), $u_{k}$ is itself a function of the state $x_{k}$, which in turn carries the effect of past disturbances propagated through the feedback loop. As a consequence, the regressor becomes correlated with the noise term $\eta_{k}$, and the orthogonality condition is violated:

(21) $$\begin{array}{c} E[\phi_{k}\eta_{k}]\neq 0, \end{array}$$

(22) $$\begin{array}{c} E[\phi_{k}e_{k}]\neq 0. \end{array}$$

This violation invalidates the fundamental assumption underlying the least squares estimator in the closed-loop setting.

### 2.6. Bias Analysis

For the closed-loop data-generating scheme, this subsection characterizes the asymptotic behavior of the least-squares estimator. Consider a regression of the form $x_{k+1}=\Theta \phi_{k}+\eta_{k}$. Estimating the parameters by minimizing the squared prediction error leads to the normal equation, whose closed-form solution is

(23) $$\hat{\Theta }={\left(\sum_{k=1}^{N}\phi_{k}\phi_{k}^{T}\right)}^{-1}\left(\sum_{k=1}^{N}\phi_{k}x_{k+1}^{T}\right),$$

provided that the information matrix $\sum_{k=1}^{N}\phi_{k}\phi_{k}^{T}$ is nonsingular.

To analyze its asymptotic behavior, the expression is normalized by the number of samples, and the probability limit is taken as N→∞:

(24) $$\mathrm{plim}\hat{\Theta }={\left(\mathrm{plim}\;\frac{1}{N}\sum_{k=1}^{N}\phi_{k}\phi_{k}^{T}\right)}^{-1}\left(\mathrm{plim}\;\frac{1}{N}\sum_{k=1}^{N}\phi_{k}x_{k+1}^{T}\right),$$

or, in compact form,

(25) $$\mathrm{plim}\;\hat{\Theta }=R_{\phi \phi }^{-1}R_{\phi x^{+}}.$$

Substituting the system dynamics relates $R_{\phi x^{+}}$ to the true parameters. Multiplying the system equation by $\phi_{k}$ and averaging over the dataset yields

(26) $$\frac{1}{N}\sum_{k=1}^{N}\phi_{k}x_{k+1}^{T}=\frac{1}{N}\sum_{k=1}^{N}\phi_{k}\phi_{k}^{T}\Theta^{T}+\frac{1}{N}\sum_{k=1}^{N}\phi_{k}\eta_{k}^{T},$$

so that, in the limit,

(27) $$R_{\phi x^{+}}=R_{\phi \phi }\Theta^{T}+R_{\phi \eta }.$$

Since $R_{\phi \eta }\neq 0$, as established in the previous subsection, it follows that

(28) $$\mathrm{plim}\;\hat{\Theta }=\Theta^{T}+R_{\phi \phi }^{-1}R_{\phi \eta }\neq \Theta^{T}.$$

The estimator is therefore asymptotically inconsistent: even with an unbounded number of samples, the estimate does not converge to the true parameters, and the asymptotic bias is given by $R_{\phi \phi }^{-1}R_{\phi \eta }$.

It is worth stressing that this bias does not require the disturbance $\eta_{k}$ to be white. In practical sensor-based acquisition, measurement noise is typically colored by anti-aliasing filters, finite sensor bandwidth, and the sampling process itself. Coloring alters the specific value of $R_{\phi \eta }$ but not the fact that it remains nonzero under feedback; consequently, the bias persists and may even grow when the noise spectrum overlaps the excited dynamics. The closed-loop bias analyzed here is thus a structural property of feedback identification from real sensor data, rather than an artifact of an idealized white-noise assumption.

For Takagi–Sugeno models the effect is amplified: by construction, several components of the regressor depend on the same state weighted by different activation functions. This introduces an additional correlation that couples all submodels simultaneously, degrading both the local and the global consistency of the estimates.

## 3. Materials and Methods

The analysis in the previous section reveals not only bias caused by closed-loop identification—it leads to a global estimation structure, where every sample influences every parameter indiscriminately. In contrast, Takagi–Sugeno models are local by nature in that each submodel is responsible for the dynamics of a specific region of state space; thus, the data carry an intrinsic non-homogeneity across rules.

This inspires the redefinition of the problem in terms of local predictions, where each sample is weighted according to its profile. The outcome is an identification method which corresponds with the Takagi–Sugeno form.

It is based on two complementary weightings from the activations of the rule. The first acts within the data: every sample is weighted by its degree of membership $\mu_{i}\left(x_{k}\right)$ in the local cost function. The second regularizes based on the penalty where the strength of each rule is divided by their cumulative activation. The proposed Dual-Weighted Takagi–Sugeno Identification (DW-TSI) framework is named as such due to this combination of weights on the residuals and penalty.

### 3.1. Rule-Wise Weighted Formulation

To align the estimating process with this local structure of the Takagi–Sugeno model, it is suggested to reformulate the identification problem by explicitly weighting the samples according to their activation in each fuzzy rule.

Denote the *i*-th fuzzy rules activation degree at time instant *k* as $\mu_{i}\left(x_{k}\right)$. From these membership functions, the weighting matrix of each rule is defined as

(29) $$W_{i}=\mathrm{diag}\left(\mu_{i}\left(x_{1}\right),\mu_{i}\left(x_{2}\right),\ldots ,\mu_{i}\left(x_{N}\right)\right)$$

Through this formulation, the original estimation problem is broken down into weighted subproblems, in which the relevance of each sample to a given submodel is scaled by its membership degree. This removes the coupling between submodels introduced by the classical formulation.

In our case, $W_{i}$ physically treats the data as a soft selection assigned to each rule: every sample can contribute to every submodel, but its contribution is regulated by the weight resulting from the activation of that specific sample. This allows for structured estimators that take into account coupling and bias, while maintaining the overall coherence and factored nature of fuzzy models.

### 3.2. Weighted Estimator

If every single sample was equally significant, then the intuitive criterion would just be minimizing the total least-squares error $\sum_{k=1}^{N}{∥e_{i,k}∥}^{2}$. This assumption is not consistent with the local nature of a Takagi–Sugeno model since not all samples give equal information for every rule. Therefore, the error of each sample is scaled with respect to how much it is activated $\mu_{i}\left(x_{k}\right)$ in order to define the local cost function as follows:

(30) $$J\left(\Theta_{i}\right)=\sum_{k=1}^{N}\mu_{i}\left(x_{k}\right)\;{∥x_{k+1}-\Theta_{i}\phi_{k}∥}^{2}$$

This expression allows a direct interpretation: whenever a sample activates the *i*-th rule strongly, its prediction error contributes more to the estimation of $\Theta_{i}$, while whenever its activation is low, its contribution to fitting that local submodel decreases. This leads to the utilization criterion shown above, which implements a soft data selection mechanism compatible with the distributed structure of fuzzy models.

Since $W_{i}$ is a diagonal matrix, the diagonal elements are used to weight different samples of the local fitting criterion. So, in this setup, the set of prediction errors for *i*-th rule can be represented as

(31) $$E_{i}=X^{+}-\Theta_{i}\Phi$$

where the *k*-th column of $E_{i}$ is given by $e_{i,k}=x_{k+1}-\Theta_{i}\phi_{k}$. Based on this definition, the scalar cost function can be reformulated in matrix form as

(32) $$J\left(\Theta_{i}\right)=\sum_{k=1}^{N}\mu_{i}\left(x_{k}\right)\;e_{i,k}^{T}e_{i,k}$$

Since $E_{i}$ collects all prediction errors column-wise and $W_{i}$ encodes the weighting associated with each sample, the previous summation can be equivalently expressed as

(33) $$J\left(\Theta_{i}\right)=\mathrm{tr}\left[E_{i}W_{i}E_{i}^{T}\right]$$

or

(34) $$J\left(\Theta_{i}\right)=\mathrm{tr}\left[\left(X^{+}-\Theta_{i}\Phi \right)W_{i}{\left(X^{+}-\Theta_{i}\Phi \right)}^{T}\right]$$

The operator tr(·) indicates that we are taking the trace of matrix. This set of notation is used to minimize the multivariate squared errors on weighted sums without losing its original meaning as a criterion. More specifically, $\mathrm{tr}(E_{i}W_{i}E_{i}^{T})$ exactly reconstructs the sum over the terms $\mu_{i}\left(x_{k}\right)\;e_{i,k}^{T}e_{i,k}$.

Expanding the expression yields

(35) $$\begin{array}{cc} J(\Theta_{i})=\; & \mathrm{tr}\left(X^{+}W_{i}X^{+T}\right)-\mathrm{tr}\left(X^{+}W_{i}\Phi^{T}\Theta_{i}^{T}\right)\\ & -\mathrm{tr}\left(\Theta_{i}\Phi W_{i}X^{+T}\right)+\mathrm{tr}\left(\Theta_{i}\Phi W_{i}\Phi^{T}\Theta_{i}^{T}\right). \end{array}$$

Using the rules from standard matrix calculus gives us

(36) $$\frac{𝜕J(\Theta_{i})}{𝜕\Theta_{i}}=-2X^{+}W_{i}\Phi^{T}+2\Theta_{i}\Phi W_{i}\Phi^{T}$$

which yields

(37) $$\Theta_{i}\Phi W_{i}\Phi^{T}=X^{+}W_{i}\Phi^{T}$$

Finally, if the matrix $\Phi W_{i}\Phi^{T}$ is invertible, then the closed-form solution is given by

(38) $${\hat{\Theta }}_{i}=X^{+}W_{i}\Phi^{T}{\left(\Phi W_{i}\Phi^{T}\right)}^{-1}$$

This estimator recovers the parameters of each local submodel by benefiting from the entire dataset and weighting each sample according its level of activation in the corresponding rule. This maintains the global coherence of the Takagi–Sugeno framework, whilst respecting its distributed nature, as each submodel is predominantly adjusted on information more representative to its own domain of operation.

Yet, while this formulation alleviates the structural coupling introduced by global estimation, numerical issues are still expected in case of insufficient local excitation or a sequence of ill-conditioned or even singular $\Phi W_{i}\Phi^{T}$ matrices. This limitation brings the need for adding a regularizer mechanism.

### 3.3. Structurally Adaptive Regularization

A ridge-type regularization term is added directly to the local information matrix. The weighted estimator corresponding to rule *i* is then given by

(39) $${\hat{\Theta }}_{i}=X^{+}W_{i}\Phi^{T}{\left(\Phi W_{i}\Phi^{T}+\lambda_{i}I\right)}^{-1},$$

where *I* is the identity matrix of proper dimensions and $\lambda_{i}>0$ is the regularization parameter for Rule *i*. By adding $\lambda_{i}I$, we push the eigenvalue spectrum of $\Phi W_{i}\Phi^{T}$ away from zero—this allows for better numerical conditioning, which ensures a well-defined estimate even in case of weak local excitation.

(40) $$N_{i}=\sum_{k=1}^{N}\mu_{i}\left(x_{k}\right),$$

and the rule-wise penalty is defined as

(41) $$\lambda_{i}=\frac{\lambda_{0}}{N_{i}},$$

where $\lambda_{0}>0$ is a single global tuning parameter. This results in a more severe penalty for rules with low cumulative activation and a milder penalty for those supported by more relevant information.

This choice is not unique; it is one way (interpretable or not) of adjusting the intensity of regularization relative to the degree of observed evidence for each submodel. Any other monotonically decreasing function of $N_{i}$ would yield the same result.

Thus, the scheme presents a balance between local fidelity and numerical stability; regions of the parameter space with low excitation do not generate divergent or non-physical parameter estimates. Algorithm 1 summarizes the entire procedure.
**Algorithm 1** Dual-weighted adaptively regularized TS identification**Require:** Dataset ${\left\{x_{k},u_{k}\right\}}_{k=1}^{N}$, membership functions $\mu_{i}\left(\cdot \right)$, number of rules *r*, global regularization parameter $\lambda_{0}$**Ensure:** 
Local model set ${\left\{A_{i},B_{i}\right\}}_{i=1}^{r}$  1:Construct the regressor $\phi_{k}$ and assemble Φ and $X^{+}$  2:**for** i=1 to *r* **do**  3:   Evaluate the activation levels $\mu_{i}\left(x_{k}\right)$ for k=1,…,N  4:   Build the diagonal weighting matrix $W_{i}=\mathrm{diag}\left(\mu_{i}\left(x_{1}\right),\ldots ,\mu_{i}\left(x_{N}\right)\right)$  5:   Compute the effective support $N_{i}=\sum_{k=1}^{N}\mu_{i}\left(x_{k}\right)$                                          (40)  6:   Compute the penalty $\lambda_{i}=\lambda_{0}/N_{i}$                                                                      (41)  7:   Estimate ${\hat{\Theta }}_{i}$                                                                                                        (39)  8:   Extract $A_{i},B_{i}\leftarrow {\hat{\Theta }}_{i}$  9:**end for**10:**return** ${\left\{A_{i},B_{i}\right\}}_{i=1}^{r}$

### 3.4. Theoretical Analysis

In this section, we lay out the main properties of the proposed estimator and present theoretical results giving necessary basis to its structural consistency, numerical behavior and identification limits in closed loop data settings.

**Proposition** **1.** *If the membership functions are uniform, i.e.,*

(42) $$\mu_{i}\left(x_{k}\right)=\frac{1}{r},\;\forall i,k,$$

*In this case, the proposed weighted regularized estimator reduces to the global ridge least-squares solution.*

**Proof.**  Let ${\hat{\Theta }}_{i}$ be the regularized weighted estimator of rule *i*, given by the formula

(43) $${\hat{\Theta }}_{i}=X^{+}W_{i}\Phi^{T}{\left(\Phi W_{i}\Phi^{T}+\lambda_{i}I\right)}^{-1},$$

with $W_{i}=\mathrm{diag}\left(\mu_{i}\left(x_{1}\right),\ldots ,\mu_{i}\left(x_{N}\right)\right)$, and with a uniform activation, $W_{i}=\frac{1}{r}I$, such that

(44) $${\hat{\Theta }}_{i}=\frac{1}{r}X^{+}\Phi^{T}{\left(\frac{1}{r}\Phi \Phi^{T}+\lambda_{i}I\right)}^{-1}=X^{+}\Phi^{T}{\left(\Phi \Phi^{T}+r\lambda_{i}I\right)}^{-1}.$$

This is indeed the global ridge least-squares solution with penalty $r\lambda_{i}$.    □

**Proposition** **2.** *The weighted regularized least-squares formulation allows the rule estimation to be fully decoupled: the global optimization problem breaks down into r independent subproblems, one per rule.*

**Proof.**  Consider the global regularized cost

(45) $$J\left(\left\{\Theta_{i}\right\}\right)=\sum_{i=1}^{r}\left(\mathrm{tr}\;\left[\left(X^{+}-\Theta_{i}\Phi \right)W_{i}{\left(X^{+}-\Theta_{i}\Phi \right)}^{T}\right]+\lambda_{i}\;\mathrm{tr}\left(\Theta_{i}\Theta_{i}^{T}\right)\right).$$

It follows that each summand is a function of $\Theta_{i}$ only, with no cross-term involving $\Theta_{j}$ for j≠i. The cost can therefore be minimized separately for each rule. Setting $𝜕J/𝜕\Theta_{i}=0$ gives

(46) $$-2X^{+}W_{i}\Phi^{T}+2\;\Theta_{i}\Phi W_{i}\Phi^{T}+2\lambda_{i}\Theta_{i}=0.$$

This gives the local normal equation

(47) $$\Theta_{i}\left(\Phi W_{i}\Phi^{T}+\lambda_{i}I\right)=X^{+}W_{i}\Phi^{T}.$$

Each parameter matrix $\Theta_{i}$ is thus obtained independently of the others, completing the proof.    □

Such decoupling property enables retaining the local interpretability of each Takagi–Sugeno rule, while avoiding a spurious coupling between submodels (an important aspect for guaranteeing that the identified system has physically plausible behavior).

**Proposition** **3.** *Let $H_{i}=\Phi W_{i}\Phi^{T}$ be the local information matrix of rule i. For any $\lambda_{i}>0$, the regularized matrix $H_{i}^{\mathrm{reg}}=H_{i}+\lambda_{i}I$ is positive definite, and its condition number satisfies $\kappa \left(H_{i}^{\mathrm{reg}}\right)\leq \kappa \left(H_{i}\right)$, with strict inequality whenever $H_{i}$ is not already perfectly conditioned.*

**Proof.**  As $W_{i}$ is diagonal with nonnegative entries, $H_{i}=\Phi W_{i}\Phi^{T}$ is symmetric and positive semidefinite, so $\lambda_{\min}\left(H_{i}\right)\geq 0$. By the eigenvalue shift property,

(48) $$\lambda_{\min}\left(H_{i}^{\mathrm{reg}}\right)=\lambda_{\min}\left(H_{i}\right)+\lambda_{i}\geq \lambda_{i}>0,$$

and hence, $H_{i}^{\mathrm{reg}}$ is positive definite and invertible. Its condition number is

(49) $$\kappa \left(H_{i}^{\mathrm{reg}}\right)=\frac{\lambda_{\max}\left(H_{i}\right)+\lambda_{i}}{\lambda_{\min}\left(H_{i}\right)+\lambda_{i}}.$$

Define $g\left(t\right)=\frac{\lambda_{\max}\left(H_{i}\right)+t}{\lambda_{\min}\left(H_{i}\right)+t}$ for t≥0. Its derivative,

(50) $$g^{\prime }\left(t\right)=\frac{\lambda_{\min}\left(H_{i}\right)-\lambda_{\max}\left(H_{i}\right)}{{\left(\lambda_{\min}\left(H_{i}\right)+t\right)}^{2}}\leq 0,$$

shows that *g* is non-increasing. Since $\lambda_{i}>0$, $g\left(\lambda_{i}\right)\leq g\left(0\right)=\lambda_{\max}\left(H_{i}\right)/\lambda_{\min}\left(H_{i}\right)=\kappa \left(H_{i}\right)$ such that if $\lambda_{\max}\left(H_{i}\right)>\lambda_{\min}\left(H_{i}\right)$, then the inequality is strict. If $H_{i}$ is singular, then $\kappa (H_{i})=\infty$ while $\kappa (H_{i,\mathrm{reg}})$ is finite so the improvement is trivial. In all examples, regularization does not degrade—and typically improves—the numerical conditioning.    □

This result is important in the case of weakly activated regions when only a few samples activate rule *i*, making $H_{i}$ near singular, and increasing $\lambda_{i}$ restores invertibility via the adaptive penalty, providing stable estimates of local parameters.

**Remark** **1** (Structural attenuation of feedback correlation)**.** *Within each local subproblem, the contribution of sample k to the regressor/disturbance cross-correlation is scaled by $\mu_{i}\left(x_{k}\right)\in \left[0,1\right]$. Thus, samples drawn from operating neighborhoods where rule i is weakly active (exactly those whose feedback-induced correlation is least relevant to submodel i) are less weighted, while each submodel is estimated on the data most representative of its own region. This form of rule-wise weighting does not abolish the closed-loop correlation, since the dependency between $x_{k}$ and $\eta_{k}$ remains present in the active region, but only localizes this effect so that it does not get transmitted across submodels as in global least squares. Every sample therefore contributes to rule i in exact proportion to its degree of membership. We empirically evaluate the amount of bias reduction achieved in Appendix A and with a sensor noise injection experiment in Section 4.*

Rule-wise weighting thus takes advantage of the local Takagi–Sugeno dynamics to bound feedback-induced correlation without requiring instrumental variables or more sophisticated closed-loop identification approaches. In combination with the adaptive regularization of Equation (41), this renders the proposed framework uniquely appropriate to settings in which only closed-loop sensor data are at hand.

## 4. Results

The DW-TSI framework is evaluated on a real aero-pendulum, which is an open-loop unstable platform and for which the data are only collected during closed-loop operation. In this context, we compare three identification methods (classical TS, N4SID, and DW-TSI) over three validation levels (one-step-ahead prediction, multi-step autonomous simulation, closed-loop applicability). A NARX neural model is also investigated here, but as shown below, is excluded after the one-step-ahead stage due to its unbounded behavior in autonomous simulation. A schematic view of the entire experimental workflow including closed-loop data acquisition and model validation is illustrated in Figure 1.

### 4.1. Aero-Pendulum Platform and Fuzzy Partition

#### 4.1.1. Experimental Platform

The aero-pendulum is a physical laboratory device with a constant carbon rod length of L=0.45 m and mass m=0.31 kg, rigidly attached at the frictionless pivot and actuated at one end by a brushless DC motor coupled to a propeller. All physical parameters of the platform are described in Table 1. The nonlinear dynamics are governed by

(51) $$J\;\ddot{\theta }\left(t\right)=K_{d}\;u^{2}\left(t\right)-b\;\dot{\theta }\left(t\right)-mgL\sin\theta \left(t\right),$$

where u(t)∈[0,1] is the normalized motor input, $K_{d}=L\;C_{f}\;K_{\omega }^{2}$ is the aerodynamic thrust coefficient, and $K_{\omega }=\frac{2\pi }{60}\;k_{v}\;V_{\mathrm{bat}}$ is the no-load angular velocity of the propeller. The system is open-loop unstable about the upright equilibrium $\theta_{0}=\pi /2$ rad ($90^{\circ }$); without feedback, any perturbation leads to an unbounded response.

All identification and validation experiments were conducted on the physical hardware. A discrete-time LQR controller with gains K=[1.0000,0.9201] and gravity compensation $d_{0}=0.5977$ maintains the pendulum near the upright equilibrium throughout every experiment (the basis for $d_{0}$ and *K* is given in Section 4.2). Working variables are expressed as deviations from equilibrium: $\tilde{\theta }=\theta -\theta_{0}$, $\tilde{\omega }=\dot{\theta }$, $\tilde{u}=u-d_{0}$.

The angular position is detected using an analog rotary potentiometer attached to the pendulum shaft. It is a standard, low-cost commercial component of no special metrological grade: its usable travel simply covers the operating range of the pendulum, and its output is read directly by the acquisition system, without any amplification or instrumentation stage, so that the effective angular resolution is set by the acquisition quantization rather than by a precision sensor. An RC circuit minimizes high-frequency pickup, and no instrumentation, precision, or anti-aliasing filter is used. The angular velocity is numerically estimated from the measured position, acquired at $f_{s}=100$ Hz, as the derivative of the potentiometer output. Therefore, the raw signals exhibit noise characteristics typical of inexpensive analog sensors, such as quantization noise, cursor terminal noise, and a noise term related to feedback from frictional movements. Thus, the platform provides a realistic framework for evaluating identification from noisy sensor data. It is worth emphasizing which quantities are measured and which are computed: only the angular position $\tilde{\theta }$ is a directly measured variable, whereas the angular velocity ω is a numerically computed quantity obtained by differentiating the measured position, and the deviation input $\tilde{u}$ is the known command applied to the motor. This distinction is reflected in the identification regressor $\phi_{k}=\left[{\tilde{\theta }}_{k},\;\omega_{k},\;{\tilde{u}}_{k}\right]$.

The *Identification* dataset comprises $N_{\mathrm{id}}=5000$ samples (50 s at $T_{s}=0.01$ s) from the physical platform with LQR stabilization and perturbation signals. Consequently, the identification data already include the inherent noise of the analog sensor. An independent $N_{\mathrm{val}}$ = 10,001 sample (100 s) validation dataset was recorded using the same controller but richer multifrequency excitation signal—a combination of sinusoidal sweeps, step sequences and broadband noise—to probe for operating conditions that were not present during identification. In all experiments, the scaling of the excitation amplitude was based on avoiding pushing any actuator into saturation.

TS rules are produced using Gaussian membership functions at centers defined for $\tilde{\theta }$ as $\left\{-30^{\circ },0^{\circ },30^{\circ }\right\}$ and $\tilde{\omega }$ as {−5, 0, 5 rad/s} 3 × 3 rule partition of the state space. The identification of the DW-TSI model does not include an affine term in line with the deviation-variable formulation for which by construction, the equilibrium point is at the origin.

For all rules and tests, the regularization parameter was set to $\lambda_{0}=1\times 10^{-3}$, chosen due to its aptitude at trading off local fidelity with the numerical stability of weakly excited rules.

#### 4.1.2. One-Step-Ahead Prediction

Table 2 presents the RMSE, MAE and fitted normalized index (Fit%) results for OSA prediction on the validation dataset, where the measured state x(k) and input u(k) is fed back into every step to avoid prediction error accumulation.

This basic test is satisfied by all four models, but the classical TS model achieves the highest fit indices (${\mathrm{Fit}}_{\tilde{\theta }}=99.92\%$, ${\mathrm{Fit}}_{\tilde{\omega }}=99.0\%$). Caution is warranted when interpreting this result: because the measured state is given at each timestep, prediction errors cannot accumulate; thus, even models with local matrices whose eigenvalues lie outside of the unit disc can perform well at this stage [6,8]. As we show in the next subsection, unregularized closed-loop TS estimation causes an observable structural instability only over longer horizons.

Both N4SID and DW-TSI accuracies across one-step errors, ${\mathrm{RMSE}}_{\tilde{\theta }}\approx 2.1\times 10^{-4}$ rad for angles and ${\mathrm{RMSE}}_{\omega }=2.79\times 10^{-3}$ rad/s for angular speeds, were almost identical, confirming that both models capture the local closed-loop dynamics very accurately on unseen data. As for the fit, DW-TSI gives a marginally better $\tilde{\theta }$ (99.74% vs. 98.10%) and ω (95.83% vs. 87.95%) compared with N4SID. While NARX achieves intermediate accuracy (${\mathrm{Fit}}_{\tilde{\theta }}=59.4\%$), it uses a correspondingly short delay window ($n_{a}=n_{b}=2$, or 20 ms of input–output history), which cannot capture the slow angle dynamics needed to represent the slower part of the system.

Although OSA performance is a necessary condition for model quality, it is not sufficient: any model with sufficiently local structure will perform well when reset to the true state at each step. Next, we present the discriminating evaluation.

#### 4.1.3. Multi-Step Autonomous Simulation

One-step-ahead prediction does not capture a model’s ability to reproduce system trajectories over extended horizons. To assess this property, each model is evaluated in autonomous simulation: the measured input $\tilde{u}\left(k\right)$ from three independent physical experiments is applied to the model, which propagates its own predicted state forward without any external state correction. The three experiments were conducted on the real hardware with the LQR controller active: an **impulse** (brief perturbation exciting the free transient response), a **sinusoidal** input at 0.5 Hz, and a **band-limited noise** sequence. Each experiment lasts 10 s at $T_{s}=0.01$ s.

Despite its acceptable OSA performance, the NARX model produces unbounded trajectories within the first few seconds of autonomous simulation across all tested configurations, including a dedicated closed-loop retraining using narxnet with output feedback. This behavior reflects a fundamental limitation of black-box neural network models in closed-loop identification: the absence of structural stability constraints causes error amplification across multi-step horizons [6]. NARX is therefore excluded from the figure and from the remaining analysis.

The results for the three remaining models can be seen in Figure 2 and Table 3. The **classical TS** model fails in all three experiments (dashed traces in Figure 2), despite scoring well on various one-step-ahead metrics, and hence is not reported among the respective error metrics in Table 3. This divergence reveals an underlying structural instability in the unregularized closed-loop estimates which had remained hidden due to one-step-ahead prediction. **N4SID** works well under impulse and noise excitation, but diverges in the angle channel under sinusoidal excitation ($\tilde{\theta }\to -0.6$ rad), demonstrating single operating-point linearization limitations at sustained higher amplitude oscillations. The only model that is bounded at all times in all scenarios, even when the reference moves away, is **DW-TSI**. In the angle channel, it achieves ${\mathrm{RMSE}}_{\tilde{\theta }}=1.15\times 10^{-3}$ rad with impulse excitation, outperforming N4SID. For sinusoidal and noise excitation, $\tilde{\theta }$ and ω are reproduced with the correct phase and frequency, even though moderate amplitude attenuation occurs for the sinusoidal case as a consequence of the conservative bias introduced by rule-wise regularization, which trades gain fidelity for guaranteed stability.

#### 4.1.4. Closed-Loop Applicability

The multi-step results establish that DW-TSI remains bounded in autonomous simulation, but do not address whether the identified model is directly usable for control design. This experiment examines that question by applying the same discrete-time LQR originally designed for the nonlinear plant to the DW-TSI model under three push disturbances of increasing magnitude: $\Delta \theta_{0}\in \left\{0.10,0.30,0.60\right\}$ rad ($6^{\circ }$, $17^{\circ }$, and $34^{\circ }$). No additional tuning is performed.

The system is stabilized in Figure 3, which represents the behavior of the DW-TSI model for all three experiments. For small and medium pushes, its recovery trajectories are indistinguishable from those of the nonlinear reference. The transients in ω between the DW-TSI and the reference system for the large push ($34^{\circ }$) last until around 0.5 s, with DW-TSI having marginally higher angular velocity at first. Nonetheless, both systems followed the exact same path to steady state within 4 s of operation, as expected; since rule-wise regularization creates only mild gain mismatches that have well-established stability vs. fidelity trade-offs in feedback control design. Lastly, the design of rule-specific controllers via parallel distributed compensation (PDC) will be explored further in future work to minimize this mismatch even more.

These results not only show that the DW-TSI model is an able predictor of system dynamics, but also that it can directly represent the plant for model-based control without requiring a new identification or the retuning of controllers. This property is a direct consequence of the structural regularization: by constraining the identification to yield local submodels that are numerically stable, DW-TSI provides identified models compatible with standard linear control design performed locally on every rule.

#### 4.1.5. Robustness to Sensor Noise

The previous results used raw potentiometer readings, which have the analog noise in a very native form. A study with injections of sensor noise was performed to test the prediction response from both identification techniques as measurement quality deteriorates further. As for the measured angle θ, it was perturbed with zero-mean Gaussian noise of a standard deviation $\sigma_{e}$ varying from $10^{-4}$ to $10^{-2}$ rad. Since angular velocity is derived by numerically differentiating the measured angle, the injected noise corrupts both regressor and target, thus reproducing exactly the correlation between regressor and error that motivates the framework. For each noise level, the estimation was performed using 20 different independent noise realizations, and the obtained models were tested in multi-step autonomous simulation with clean validation datasets. The significance of this study is that the recorded input was not re-generated through the controller; hence, it isolates errors-in-variables coupling in the data recorded rather than full loop noise propagation.

The results are presented in Figure 4 through two complementary indicators, namely the median multi-step fit index (a measure of predictive accuracy) and the parametric drift ${\mathrm{mean}}_{i}\;{∥A_{i}\left(\sigma \right)-A_{i}\left(0\right)∥}_{F}$ (how far identified local matrices move away from their noise-free reference). Panel (a) presents the classical TS model, which fails catastrophically: at $\sigma_{e}=10^{-4}$ rad, its fit index hits the saturation floor and with it, the models are rendered useless for autonomous simulation. The DW-TSI framework being proposed provides an equally good fit to about one more order of noise, and does so in a gradual fashion rather than catastrophically. Panel (b) shows the same trend for the parameters: with increasing noise, the local matrices of the classical model drift by almost three orders of magnitude away from their true (noise-free) values; in contrast, the regularized submodels stay closer to their corresponding true values.

Table 4 reports the corresponding figures. The table footnote provides a summary of the number of diverging realizations, cases in which the identified model exploded during autonomous simulation. Both estimators are virtually unaffected by this at the lowest injected level. At the same time, though, classical estimates become rapidly less reliable beyond this point: at $\sigma_{e}=10^{-3}$ rad (the saturation limit of the fit index), the local matrices also drifted by nearly three orders of magnitude from their noise-free reference, and divergences between individual realizations begin to appear. On the contrary, DW-TSI has no divergence, retains a positive fit at all levels and a parametric drift less than 1.6 in the whole range. Overall, these findings validate that the combination techniques of dual weighting and adaptive regularization from the proposed framework results in a clear robustness benefit for identification from noisy sensor measurements.

### 4.2. Simulation Study: Model Validation, Parameter Sensitivity, and Regularization Tuning

To complement the single physical platform with a controllable ground-truth reference, a nonlinear simulator of the aero-pendulum was built from the model in Equation (51) using the parameters of Table 1. The continuous dynamics are integrated with a fourth-order Runge–Kutta scheme at $T_{s}=0.01$ s, and the measurement chain of the physical device is reproduced—potentiometer quantization, additive zero-mean noise, and RC-filtered numerical differentiation for the angular velocity—so that identification is carried out on synthetic closed-loop data sharing the noise characteristics of the hardware. This setup enables a direct comparison between simulation and experiment under known parameters, a sensitivity analysis with respect to the physical parameters, and a systematic study of the regularization parameter $\lambda_{0}$.

#### 4.2.1. Design Basis and Simulation-to-Experiment Consistency

The two controller constants used throughout the experimental campaign follow from the plant model rather than from ad hoc tuning. The gravity compensation $d_{0}$ is the stationary input that balances the gravitational torque at the upright equilibrium; imposing $\ddot{\theta }=\dot{\theta }=0$ in Equation (51) gives $K_{d}\;d_{0}^{2}=mgL\sin\theta_{0}$, hence

(52) $$d_{0}=\sqrt{\frac{mgL\sin\theta_{0}}{K_{d}}}=0.5977,$$

which coincides with the value listed in Table 1. The stabilizing gain K=[1.0000,0.9201] is obtained from a discrete-time LQR applied to the model linearized about $\left(\theta_{0},0,d_{0}\right)$; with weights Q=diag(0.89,0.93) and R=0.27 the discrete LQR returns K=[0.9919,0.9283], reproducing the experimental gains. Note that at $\theta_{0}=\pi /2$ the linearized restoring term $-mgL\cos\theta_{0}$ vanishes, so the plant is marginally unstable about the upright position and locally unstable for $\tilde{\theta }>0$; the identified autonomous submodels therefore lie naturally close to the unit circle, a property revisited below.

Running the complete DW-TSI pipeline on synthetic closed-loop data reproduces the experimental behavior. One-step-ahead validation yields ${\mathrm{Fit}}_{\tilde{\theta }}=98.3\%$ and ${\mathrm{Fit}}_{\omega }=94.0\%$ (Figure 5), in close agreement with the 99.7% and 95.8% measured on the hardware (Table 2); the small residual gap is consistent with the additional unmodeled effects present in the physical device. The identified model likewise remains bounded in multi-step autonomous simulation, mirroring the experimental result.

#### 4.2.2. Sensitivity to Physical-Parameter Variation

A one-at-a-time sensitivity analysis was performed by perturbing each physical parameter of Table 1 by ±20% about its nominal value, regenerating the closed-loop data, and re-identifying the DW-TSI model. Figure 6 summarizes the resulting worst-case multi-step accuracy as a tornado chart. The identification is robust to the arm length *L* and the viscous friction *b*, whose ±20% variation keeps the median angle error near its nominal value (≈$3^{\circ }$). In contrast, the model is markedly more sensitive to the mass *m* and the thrust coefficient $C_{f}$, for which the same perturbation raises the error to ≈$16^{\circ }$. This ordering is physically consistent: *m* and $C_{f}$ set the gravity-to-thrust balance and hence the operating point and the feedforward $d_{0}$, so a deviation in either shifts the closed-loop trajectory away from the region for which the controller was designed, degrading the quality of the closed-loop identification data.

#### 4.2.3. Regularization Tuning and the Weakly Excited Rule Mechanism

The choice $\lambda_{0}=10^{-3}$ was examined by sweeping the global regularization parameter over six decades, $\lambda_{0}\in \left[10^{-6},\;10^{0}\right]$, and reporting the median multi-step fit over six independent data realizations (Figure 7). The curve exhibits a clear optimum: for $\lambda_{0}≲10^{-5}$, the penalty is negligible and the estimator reproduces the poorer behavior of the unregularized solution, whereas for $\lambda_{0}≳10^{-1}$, the local submodels are over-shrunk and accuracy collapses. A broad near-optimal plateau spans $10^{-4}$–$10^{-3}$, which justifies the selected value and provides a practical tuning guideline: $\lambda_{0}$ should be large enough to stabilize weakly excited rules yet small enough to leave well-supported rules essentially unpenalized.

The mechanism underlying this behavior is made explicit in Figure 8, which reports the spectral radius $\max_{i}\left|\lambda \left(A_{i}\right)\right|$ of every local state-transition matrix for the classical (unregularized) and the DW-TSI estimators. For the well-excited central rules, the two estimators coincide, in agreement with the Proposition of Section 3: when a rule accumulates sufficient activation, its adaptive penalty $\lambda_{i}=\lambda_{0}/N_{i}$ becomes negligible and DW-TSI reduces to the global ridge solution. The difference emerges precisely at the weakly excited corner rules (high $|\tilde{\theta }|$ combined with high |ω|), which are visited only rarely during closed-loop operation: there the unregularized estimates sit on the stability boundary (|λ|≈1), so the corresponding submodels diverge over long horizons, whereas the adaptive penalty—large for small $N_{i}$—contracts their eigenvalues well inside the unit circle (|λ|≈0.6–0.8), guaranteeing bounded local models. This confirms, on a controlled ground-truth model, that the advantage of DW-TSI is structurally localized to the weakly excited regions, exactly as observed on the physical platform.

## 5. Discussion

The aero-pendulum has been widely used as a benchmark for nonlinear and fuzzy control, including Takagi–Sugeno modeling and identification approaches [59,60,61]. Relative to those works, which identify the local models from dedicated experiments and focus primarily on control design, the present study performs the identification entirely from closed-loop, low-cost sensor data and explicitly addresses the resulting regressor–disturbance correlation; the simulation results in Section 4.2 further confirm that the reported behavior is reproduced under known parameters.

The results over the three validation levels look consistent across all dimensions. One-step-ahead prediction (Table 2) is only a necessary condition; while both N4SID and DW-TSI perform very well at this stage, and the classical TS model attains the best fit indices, neither NARX nor the classical TS model could survive autonomous simulation. This discriminating evidence is drawn from the multi-step and closed-loop experiments, where DW-TSI is not only bounded under every excitation but also directly usable for LQR design without any retuning compared to other methods.

The comparison with N4SID is framed in terms of a tie instead of better accuracy. While N4SID is a solid linear benchmark, it provides one black-box state-space realization with no actual physical interpretation of its internal states. Unlike the former, DW-TSI essentially keeps a bank of interpretable local submodels corresponding to the operating regions of the plant. In fact, the benefit of such a framework is to achieve predictively accurate results that are still local enough in the input space to allow for model-based control on par with N4SID. This limitation is evidenced by the drift of N4SID in the angle channel when under consistent sinusoidal excitation, demonstrating the limitation of a single operating-point linearization relative to a rule-based representation.

Regularization exhibits a classic division between attenuation of local gains and local estimates of numerically stable bounded submodels. This attenuation manifests as a decrease in amplitude in the sinusoidal case and a small transient mismatch under strong excitation. This achieves an intentional balance between gain fidelity and stability, and it is postulated that the parallel distributed compensation (PDC) design of rule-specific controllers will mitigate the residual mismatch.

It is useful to position DW-TSI with respect to established closed-loop identification strategies. Instrumental-variable and prediction-error methods address the regressor–disturbance correlation directly, either by introducing instruments that are uncorrelated with the noise or by estimating a parameterized noise model; they are well developed in the linear setting but do not, by themselves, deliver a bank of physically interpretable local submodels. Existing regularized Takagi–Sugeno approaches, in turn, stabilize the estimation through a global ridge or smoothness penalty applied uniformly across the model. DW-TSI is distinct on two points: the correlation is not cancelled but structurally confined to the region where each rule is active (Section 3), and the penalty is made rule-dependent through the adaptive term $\lambda_{i}=\lambda_{0}/N_{i}$, so that regularization acts exactly where local excitation is weakest. Consequently, DW-TSI is complementary to instrumental-variable and prediction-error techniques—it could be combined with an instrument or an explicit noise model—while retaining the local, physically meaningful structure that those methods do not aim to preserve.

Although the framework has been demonstrated on a single-input aero-pendulum, its formulation is not specific to this plant. The estimator operates on a generic deviation-variable regressor and a partition of unity, so it extends directly to multi-input and higher-order state-space systems: each rule remains an independent weighted ridge regression the size of a single local submodel, and the rules can be solved in parallel. The main consideration when scaling to higher dimensions is the design of the fuzzy partition, since the number of rules grows combinatorially with the number of scheduling variables—a property shared by all Takagi–Sugeno representations and commonly mitigated by choosing scheduling variables parsimoniously or by data-driven partitioning. The adaptive regularization is expected to become more valuable, not less, as the dimension grows, because weakly excited rules occur more frequently in larger operating spaces.

Regarding computational cost, it is important to distinguish the identification stage from the eventual control stage. DW-TSI is an offline identification procedure, executed once and prior to controller design, so its cost is not subject to real-time constraints. Even so, it remains modest: for *r* rules, *N* samples, and a regressor of dimension p=n+m, the method assembles and inverts one p×p regularized information matrix per rule, an overall cost of order $O(r\;p^{2}N+r\;p^{3})$ that is trivially parallelizable across the independent rules; the adaptive regularization modifies only the matrix that is inverted, so DW-TSI costs essentially the same as classical rule-wise least squares while avoiding its numerical failures. The computational budget that is genuinely critical is that of the controller ultimately deployed on general-purpose embedded hardware such as microcontrollers or PLCs. Because DW-TSI yields a bank of interpretable local linear submodels, the resulting controller reduces to a membership-weighted combination of local linear control laws, which is lightweight to evaluate; a detailed real-time implementation and benchmarking of such a controller—for instance through parallel distributed compensation—is left for future work.

Two limitations should be made explicit. First, instead of removing the dependence between $x_{k}$ and $\eta_{k}$, the rule-wise weighting confines it locally to the area where each rule is active, as shown in the Remark in Section 3 and Appendix A. As such, this framework does not replace but rather complements instrumental-variable or subspace approaches to closed-loop identification. Second, this study on sensor noise injection is a post hoc analysis done on the recorded data: it does not regenerate the perturbed input with respect to the controller, thus confining errors-in-variables coupling to the recorded signals only, instead of reproducing the full propagation of noise around the loop. Future work is left to study closed-loop noise propagation.

## 6. Conclusions

This work introduces DW-TSI, a methodology based on the problem of identification of Takagi–Sugeno (TS) models from closed-loop sensor measurements, which surpasses classical least-squares limitations. In feedback, the regressors—activation-weighted combinations of the measured variables—become correlated with the disturbances. Thus, the least-squares assumption of exogeneity is violated, and this leads to biased estimates.

This framework is based on two mechanisms which complement each other. We adopt a rule-wise weighted least-squares formulation that decouples the global estimation into local subproblems maintaining physical interpretability of each state-space submodel, and an activation-conditioned adaptive regularization that scales the penalty of a given rule inversely to its effective support, yielding better numerical conditioning specifically in weakly excited regions. Theoretical analysis shows that the formulation reduces to a global ridge regression under uniform activation, completely decouples the rule-wise subproblems, and provides strict improvement of conditioning for each local information matrix.

The framework was applied to a physical aero-pendulum identified solely from closed-loop measurements. DW-TSI matched N4SID’s accuracy for one-step-ahead prediction and remained the only approach to be bounded across all multi-step autonomous simulations, while being shown to be directly deployable for LQR-based control without any retuning. Finally, measurement noise was found to gradually drop the accuracy of DW-TSI without diverging, while unregularized TS estimation collapsed. This validates the efficacy of the proposed framework for identification from real sensor data.

Future work will include both the design of rule-specific controllers using parallel distributed compensation (PDC) to minimize residual gain mismatch introduced by the regularization, and adaptation of this framework to a complete closed-loop noise-propagation setting.

## Figures and Tables

**Figure 1:**
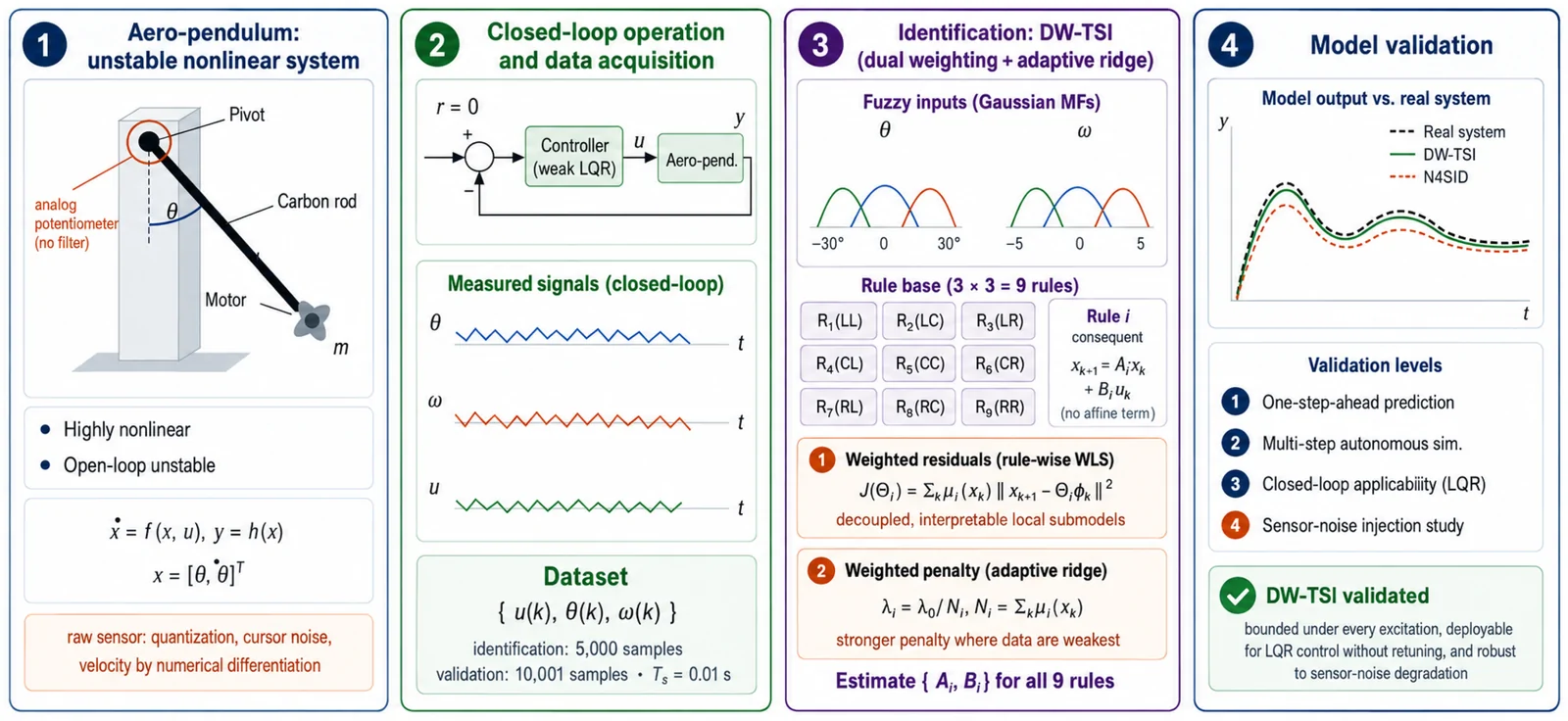
Closed-loop identification workflow for the aero-pendulum. (**1**) The open-loop–unstable platform is held near the upright equilibrium by an LQR controller. (**2**) Closed-loop data are acquired under weak excitation: only the angular position $\tilde{\theta }$ is measured, the angular velocity ω is obtained by numerical differentiation of the measured position, and the motor command $\tilde{u}$ is recorded. (**3**) A nine-rule Takagi–Sugeno model is identified with the proposed DW-TSI estimator. (**4**) The identified model is validated on independent data through one-step-ahead prediction, multi-step autonomous simulation, and closed-loop tests.

**Figure 2:**
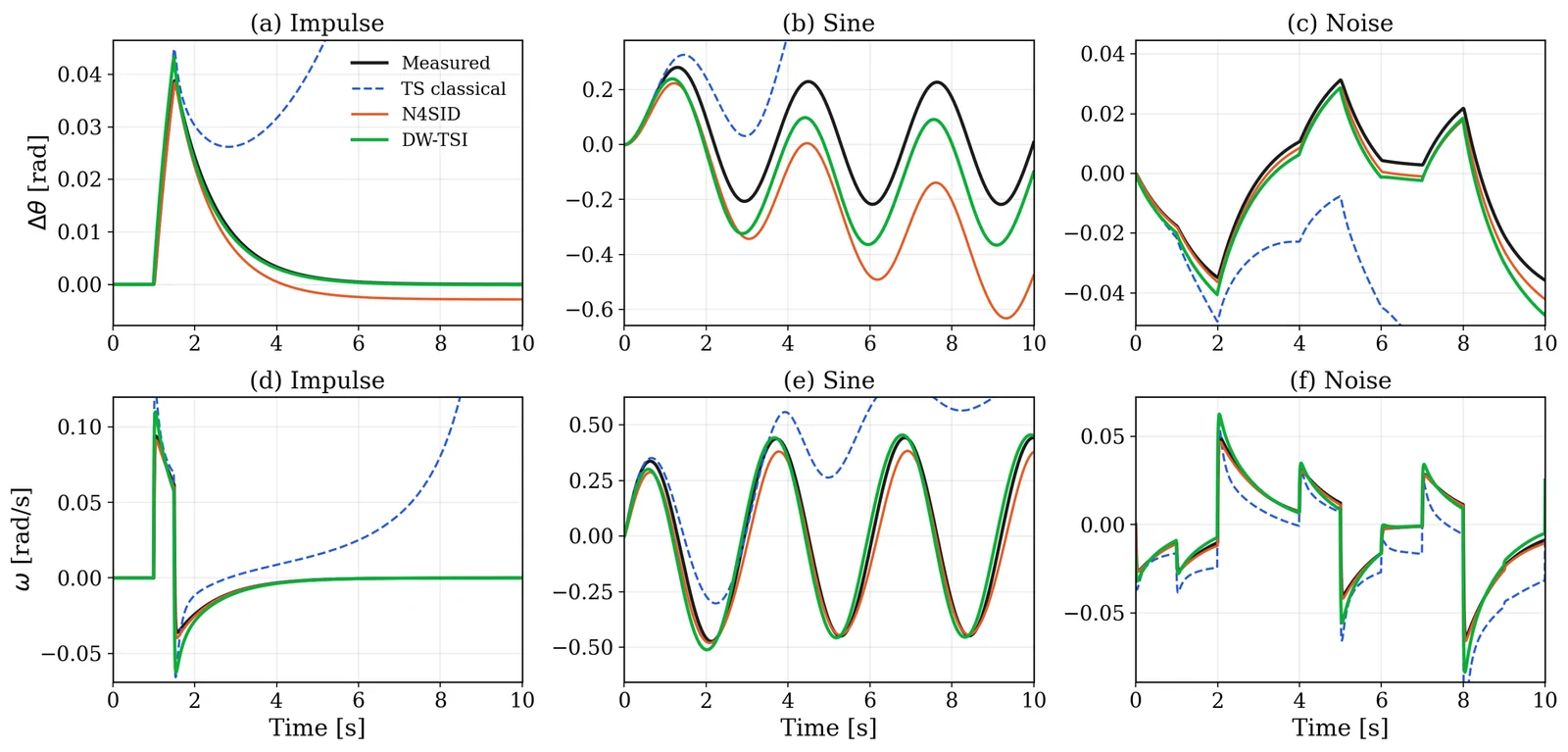
Multi-step autonomous simulation on three physical experiments. Rows: angle deviation $\tilde{\theta }$ (**a**–**c**) and angular velocity ω (**d**–**f**). Columns: impulse, sinusoidal, band-limited noise. The classical TS model (dashed) diverges throughout; N4SID diverges in $\tilde{\theta }$ under sinusoidal excitation (panel (**b**)).

**Figure 3:**
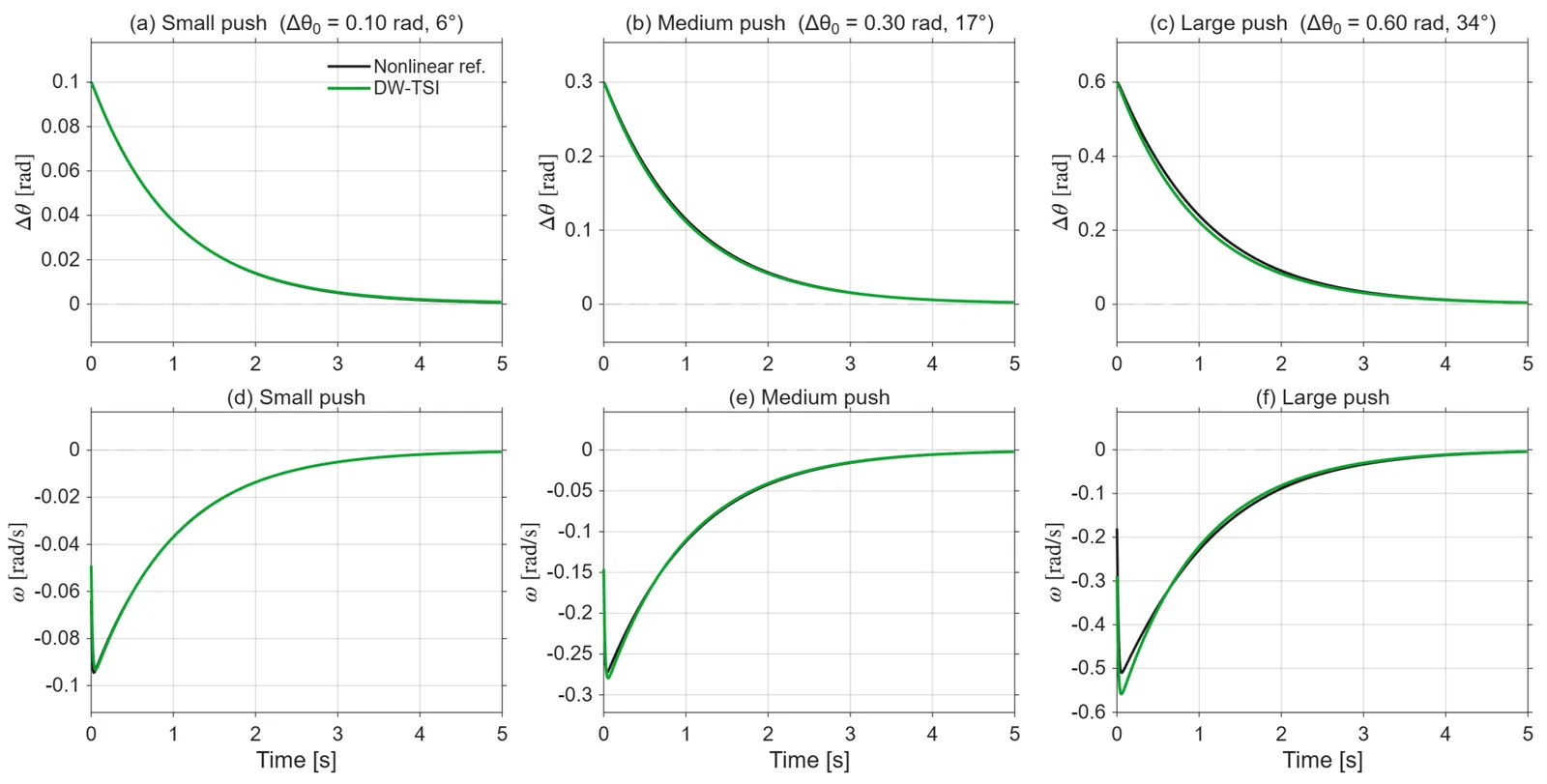
Closed-loop recovery under the LQR designed for the nonlinear plant, applied to the DW-TSI model without retuning. Push magnitudes: $\Delta \theta_{0}=0.10$ rad ($6^{\circ }$), 0.30 rad ($17^{\circ }$), 0.60 rad ($34^{\circ }$). (**a**–**c**) $\tilde{\theta }$. (**d**–**f**) ω.

**Figure 4:**
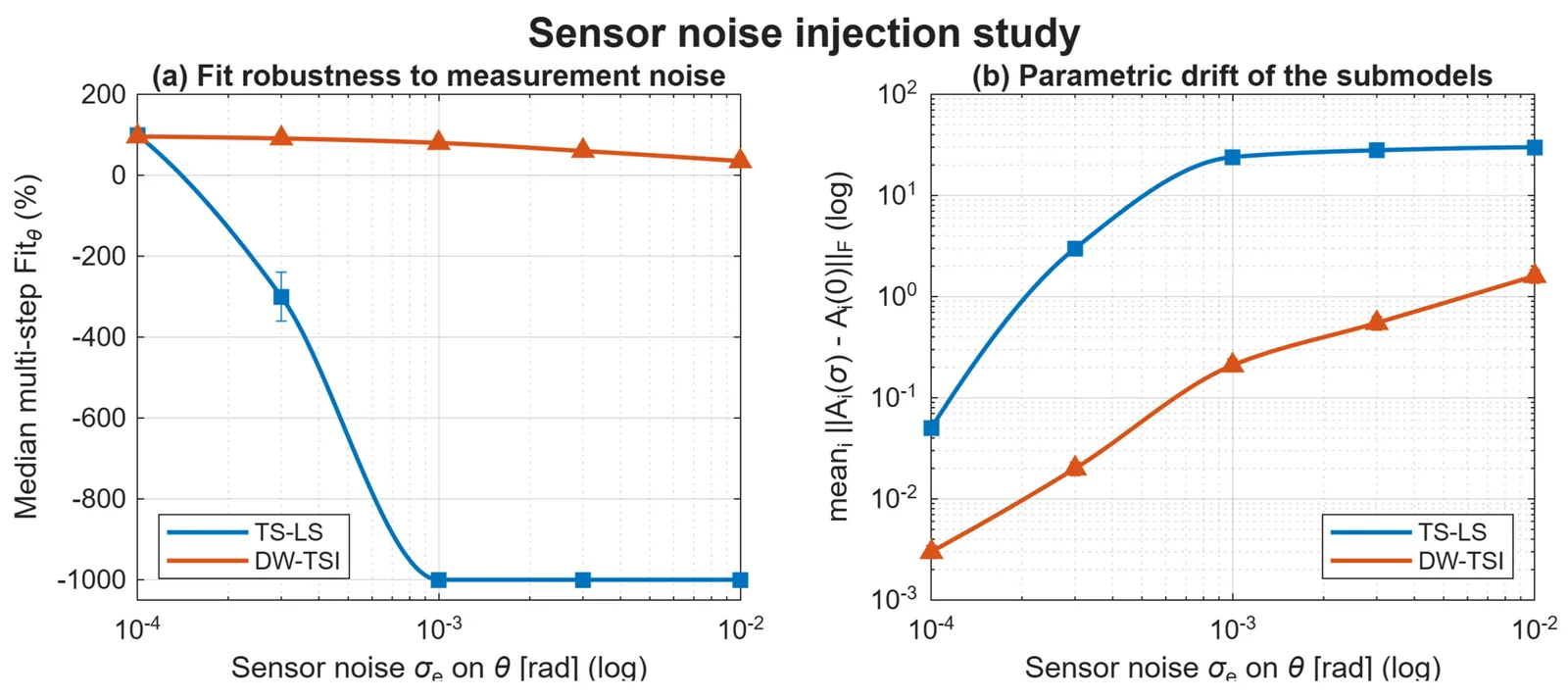
Sensor noise injection study. Median multi-step fit index (**a**) and parametric drift (**b**) versus noise level $\sigma_{e}$. TS-LS collapses beyond $\sigma_{e}=10^{-4}$ rad; DW-TSI degrades gradually.

**Figure 5:**
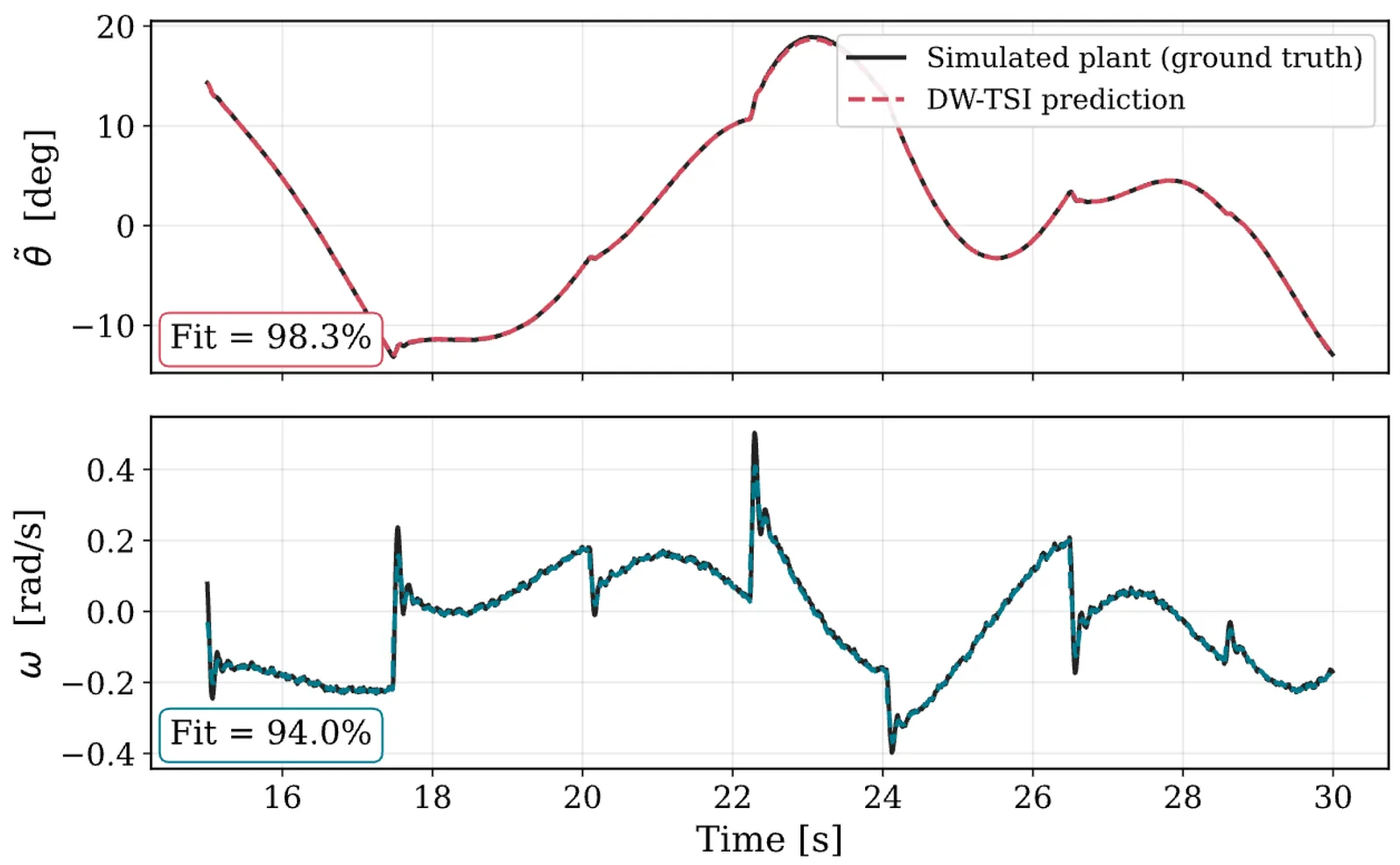
One-step-ahead validation on simulated closed-loop data with the nominal parameters of Table 1. Angle deviation $\tilde{\theta }$ (**top**) and angular velocity ω (**bottom**): simulated plant (solid) versus DW-TSI prediction (dashed). The fit indices match those obtained on the physical platform.

**Figure 6:**
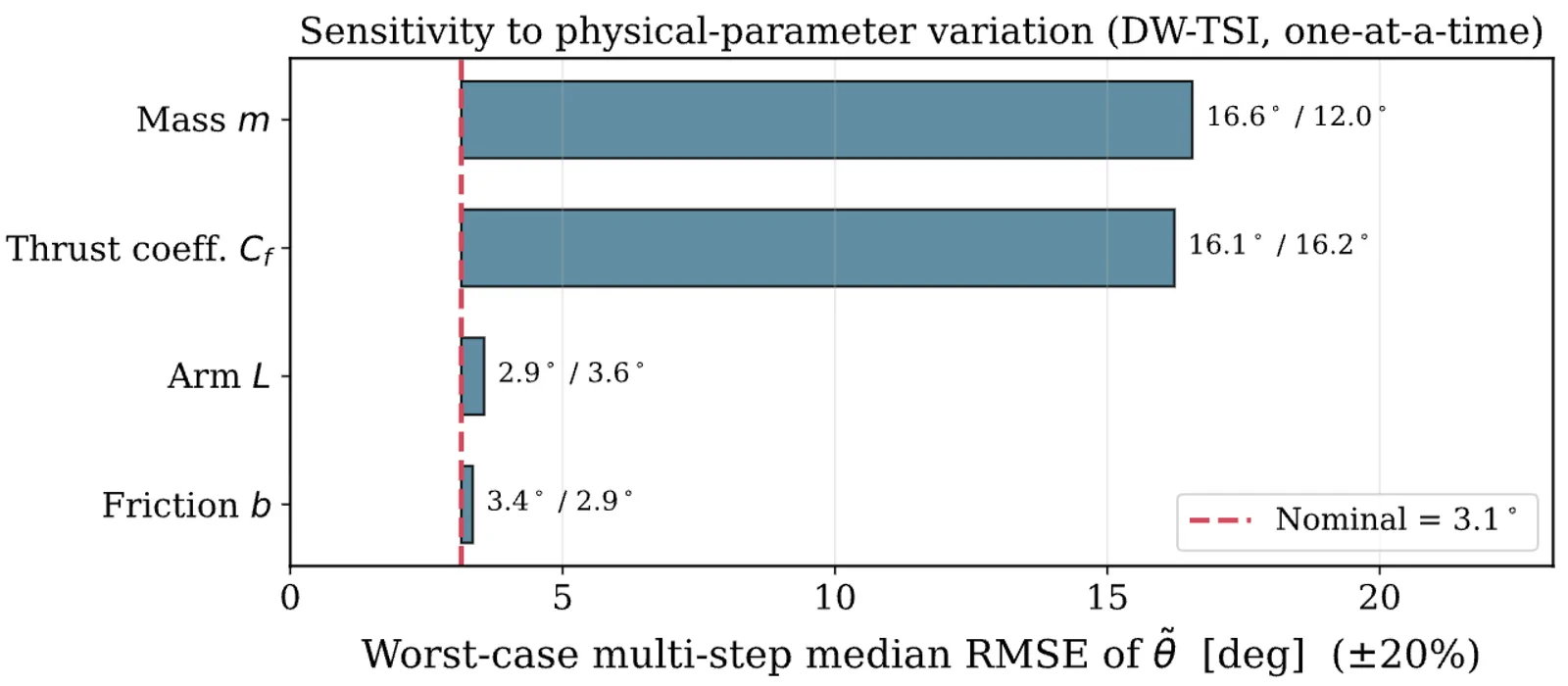
Sensitivity of the DW-TSI model to ±20% one-at-a-time variation of the physical parameters, reported as the worst-case multi-step median RMSE of $\tilde{\theta }$. The dashed line marks the nominal value; the model is most sensitive to *m* and $C_{f}$ and robust to *L* and *b*.

**Figure 7:**
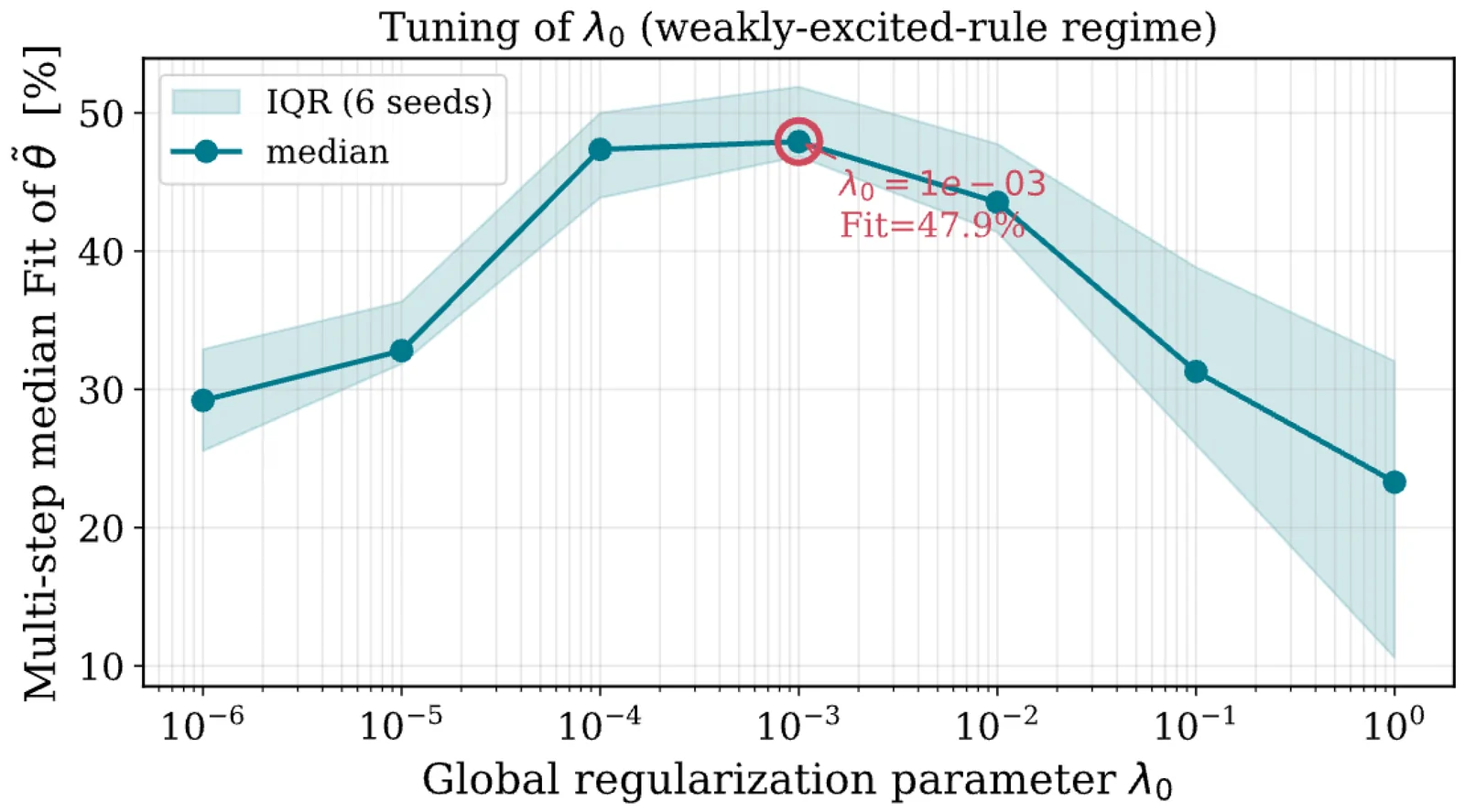
Tuning of the global regularization parameter $\lambda_{0}$: median multi-step fit of $\tilde{\theta }$ over six data realizations (shaded band: interquartile range). A near-optimal plateau spans 10^−4^–10^−3^, supporting the selected $\lambda_{0}=10^{-3}$.

**Figure 8:**
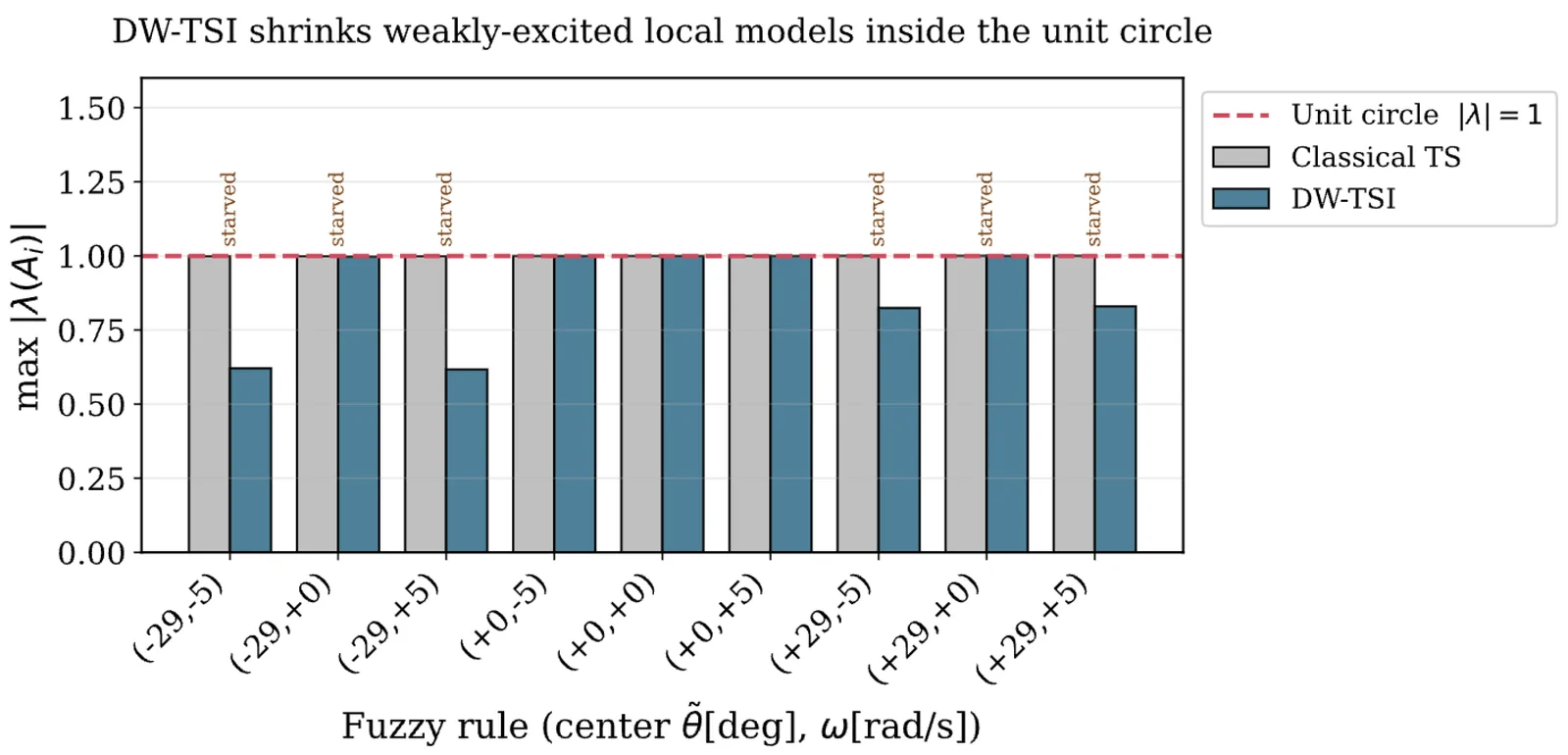
Per-rule spectral radius $\max_{i}\left|\lambda \left(A_{i}\right)\right|$ for the classical TS and DW-TSI estimators. For well-excited rules both coincide; for the weakly excited (“starved”) corner rules DW-TSI contracts the eigenvalues inside the unit circle while the unregularized estimates remain on the stability boundary.

**Table 1:** Physical parameters of the aero-pendulum platform.

Symbol	Description	Value	Unit
*m*	Mass	0.31	kg
*L*	Length	0.45	m
*b*	Viscous friction	0.45	kg/s
$J=mL^{2}$	Inertia	0.0628	kg m^2^
$C_{f}$	Aerodynamic coef.	$2.75\times 10^{-6}$	N s^2^/rad^2^
$k_{v}$	Motor constant	1400	rpm/V
$V_{\mathrm{bat}}$	Supply voltage	12	V
$d_{0}$	Gravity compensation	0.5977	–
$T_{s}$	Sampling time	0.01	s
$\tau_{\mathrm{RC}}$	Sensor RC time constant	∼0.01	s

**Table 2:** One-step-ahead prediction on the validation dataset ($N_{\mathrm{val}}$ = 10,001, $T_{s}$ = 0.01 s).

Model	${\mathrm{RMSE}}_{\tilde{\theta }}$ (rad)	${\mathrm{RMSE}}_{\omega }$ (rad/s)	${\mathrm{MAE}}_{\tilde{\theta }}$ (rad)	${\mathrm{MAE}}_{\omega }$ (rad/s)	${\mathrm{Fit}}_{\tilde{\theta }}$ (%)	${\mathrm{Fit}}_{\omega }$ (%)
TS classical	$5.67\times 10^{-4}$	$2.44\times 10^{-3}$	$5.62\times 10^{-4}$	$1.37\times 10^{-4}$	99.92	99.0
N4SID	$2.08\times 10^{-4}$	$2.79\times 10^{-3}$	$1.71\times 10^{-4}$	$1.86\times 10^{-3}$	98.10	87.95
NARX	$4.43\times 10^{-3}$	$1.92\times 10^{-3}$	$2.98\times 10^{-3}$	$1.16\times 10^{-3}$	59.37	91.72
DW-TSI	$2.09\times 10^{-4}$	$2.79\times 10^{-3}$	$1.72\times 10^{-4}$	$1.86\times 10^{-3}$	99.74	95.83

**Table 3:** Multi-step autonomous simulation: RMSE and MAE on three physical validation experiments. Classical TS and NARX diverge throughout; N4SID diverges in $\tilde{\theta }$ under sinusoidal excitation. Best stable values per experiment in **bold**.

Experiment	Model	${\mathrm{RMSE}}_{\tilde{\theta }}$ (rad)	${\mathrm{RMSE}}_{\omega }$ (rad/s)	${\mathrm{MAE}}_{\tilde{\theta }}$ (rad)	${\mathrm{MAE}}_{\omega }$ (rad/s)
Impulse	N4SID	2.52 × 10^−3^	**7.23 × 10^−4^**	2.31 × 10^−3^	**2.67 × 10^−4^**
	DW-TSI	**1.15 × 10^−3^**	5.34 × 10^−3^	**4.65 × 10^−4^**	1.44 × 10^−3^
Sine	N4SID	2.70 × 10^−1^	5.98 × 10^−2^	2.35 × 10^−1^	4.92 × 10^−2^
	DW-TSI	**1.28 × 10^−1^**	**6.07 × 10^−2^**	**1.17 × 10^−1^**	**5.15 × 10^−2^**
Noise	N4SID	**3.32 × 10^−3^**	**8.45 × 10^−4^**	**2.89 × 10^−3^**	**6.05 × 10^−4^**
	DW-TSI	5.59 × 10^−3^	5.23 × 10^−3^	5.01 × 10^−3^	2.80 × 10^−3^

**Table 4:** Sensor noise injection study: median multi-step fit index and parametric drift of the local matrices versus noise level $\sigma_{e}$ (median over independent realizations). Fit values at the saturation floor are reported as ≤−10^3^. Best value per level in **bold**.

$\sigma_{e}$ [rad]	Method	${\mathrm{Fit}}_{\theta }$ [%]	Drift *A*
10^−4^	TS-LS	98	0.05
	DW-TSI	**98**	**0.003**
3 × 10^−4^	TS-LS	−300	3.0
	DW-TSI	**93**	**0.021**
10^−3^	TS-LS	≤−10^3^	24
	DW-TSI	**81**	**0.21**
3 × 10^−3^	TS-LS	≤−10^3^	29
	DW-TSI	**62**	**0.55**
10^−2^	TS-LS	≤−10^3^	31
	DW-TSI	**39**	**1.6**

The classical TS model diverges in 2–4 of the realizations for $\sigma_{e}\geq 10^{-3}$ rad; DW-TSI never diverges at any level.

## Data Availability

The experimental datasets and the accompanying identification and simulation code that support the findings of this study are available from the corresponding author upon reasonable request.

## Appendix A. Numerical Illustration of Local Correlation Attenuation

This appendix provides a numerical example of the structural mitigation presented in the Remark from Section 3. It demonstrates the way that rule-wise weighting can alleviate the correlation term entailing closed-loop bias in a local subproblem, using a simple two-rule partition.

Consider the scalar sequences

ϕ=[2,1,−1,−2],η=[1,1,−1,−1].

The classical (unweighted) correlation term is

$$\sum_{k=1}^{4}\phi_{k}\eta_{k}=2\left(1\right)+1\left(1\right)+\left(-1\right)\left(-1\right)+\left(-2\right)\left(-1\right)=6.$$

Define a two-rule partition satisfying the partition-of-unity property:

$$\mu_{1}=\left[0.5,\;0.4,\;0.1,\;0\right],\;\mu_{2}=\left[0.5,\;0.6,\;0.9,\;1\right],$$

$$\mu_{1}\left(x_{k}\right)+\mu_{2}\left(x_{k}\right)=1.$$

The weighted correlation term for rule 1 is

$$\sum_{k=1}^{4}\mu_{1}\left(x_{k}\right)\;\phi_{k}\eta_{k}=0.5\left(2\right)\left(1\right)+0.4\left(1\right)\left(1\right)+0.1\left(-1\right)\left(-1\right)+0\left(-2\right)\left(-1\right)=1.5.$$

For this partition, the weighted correlation term of rule 1 is markedly smaller in magnitude than the unweighted term:

$$\left|\sum_{k=1}^{4}\mu_{1}\left(x_{k}\right)\;\phi_{k}\eta_{k}\right|\ll \left|\sum_{k=1}^{4}\phi_{k}\eta_{k}\right|.$$

This reduction occurs because rule 1 is weakly active only at the samples which are most contributive in the global correlation, and so their local influence gets diluted as a result of the weighting. This is an illustrative rather than a general example: while the rule-wise weighting guarantees that the correlation induced by feedback will be constrained to only the submodel regions about each active rule, it does not guarantee that this will lead to a reduction across all realizations of data. This attenuation’s net effect on identification performance is quantified experimentally in the sensor noise injection benchmark in Section 4.
